# Long non-coding RNAs are involved in the crosstalk between cancer-associated fibroblasts and tumor cells

**DOI:** 10.3389/fimmu.2024.1469918

**Published:** 2024-12-09

**Authors:** Chenbo Yang, Jiao Shu, Yiwei Li, Na Zhao, Xiaonan Liu, Xiangyu Tian, Zexin Sun, Muhammad Saud Tabish, Yichen Hong, Kuisheng Chen, Miaomiao Sun

**Affiliations:** ^1^ Department of Pathology, The First Affiliated Hospital of Zhengzhou University, Zhengzhou, China; ^2^ Henan Key Laboratory of Tumor Pathology, Zhengzhou University, Zhengzhou, China; ^3^ Henan Institute of Medical and Pharmaceutical Sciences, Zhengzhou University, Zhengzhou, China

**Keywords:** long non-coding RNAs, cancer-associated fibroblasts, tumor microenvironment, signaling pathway, tumor growth

## Abstract

The proliferation of tumors is not merely self-regulated by the cancer cells but is also intrinsically connected to the tumor microenvironment (TME). Within this complex TME, cancer-associated fibroblasts (CAFs) are pivotal in the modulation of tumor onset and progression. Rich signaling interactions exist between CAFs and tumor cells, which are crucial for tumor regulation. Long non-coding RNAs (LncRNAs) emerge from cellular transcription as a class of functionally diverse RNA molecules. Recent studies have revealed that LncRNAs are integral to the crosstalk between CAFs and tumor cells, with the capacity to modify cellular transcriptional activity and secretion profiles, thus facilitating CAFs activation, tumor proliferation, metastasis, drug resistance, and other related functionalities. This comprehensive review revisits the latest research on LncRNA-mediated interactions between CAFs and tumor cells, encapsulates the biological roles of LncRNAs, and delves into the molecular pathways from a broader perspective, aspiring to offer novel perspectives for a deeper comprehension of the etiology of tumors and the enhancement of therapeutic approaches.

## 1 Introduction

According to the latest estimates by the International Agency for Research on Cancer, about one in five men or women will develop cancer in their lifetime, and approximately one in nine men and one in twelve women will die from it. Cancer remains a significant threat to human life and health (1). Over the years, clinicians and researchers have focused on the “pathological state caused by genetic changes in the tumor (Somatic Mutation Theory, SMT)” and have designed cancer treatment methods based on this theory. However, the individual differences in treatment effects compel us to consider the potential functions of the tumor stroma (2). The progression of cancer is not a single growth process of tumor cells, but an interaction based on the tumor microenvironment (TME) and tumor cells. Even if some cells have a high tumor mutation burden, there are no obvious pathological changes, indicating that the external TME can manipulate tumor cells to present different fates (3, 4). In colorectal cancer, lung cancer, breast cancer, and esophageal cancer, high stromal tumors often indicate a low survival rate, and the proportion of tumor stroma is related to prognosis (5). Cancer-Associated Fibroblasts (CAFs) are the most prominent components of the tumor stroma and have a profound impact on the process of tumor occurrence and development. They play a key regulatory role in tumor cell proliferation, metabolic reprogramming, migration and invasion, stemness, as well as the remodeling of the tumor extracellular matrix (ECM), angiogenesis, metastasis, immune suppression, and resistance to therapy (6, 7).

Although the SMT cannot fully explain the disease of cancer, cancer is still considered a “genetic disease” where extensive genetic changes within cells disrupt tissue homeostasis and promote the development of cancer. With the advancement of gene sequencing technology, the results of genetic research have increased exponentially. Approximately 75% of the human genome is transcribed, but only 2% of the transcribed genes are mRNAs that encode proteins, with the rest initially considered “transcriptional waste” (8). After further deepening the molecular understanding of cancer, it has been found that non-coding RNAs (ncRNAs) are involved in the occurrence, progression, and metastasis of cancer. In particular, long non-coding RNAs (LncRNAs), which are transcripts longer than 200 nucleotides, differ from mRNAs and miRNAs and form a rich and complex network of tumor LncRNAs due to their various mechanisms of action (9).

This article links CAFs and LncRNA in the TME, reviews recent relevant research, and focuses on LncRNA as an important participant between CAFs and tumors, its impact on tumors and related molecular networks. It explores the occurrence and development of tumors from multiple perspectives of “tumor stroma” and “genes”, which helps to deepen the understanding of cancer diseases and innovate cancer treatment models.

## 2 Insights into cancer-associated fibroblasts

### 2.1 Diversity of origins

**Figure 1:**
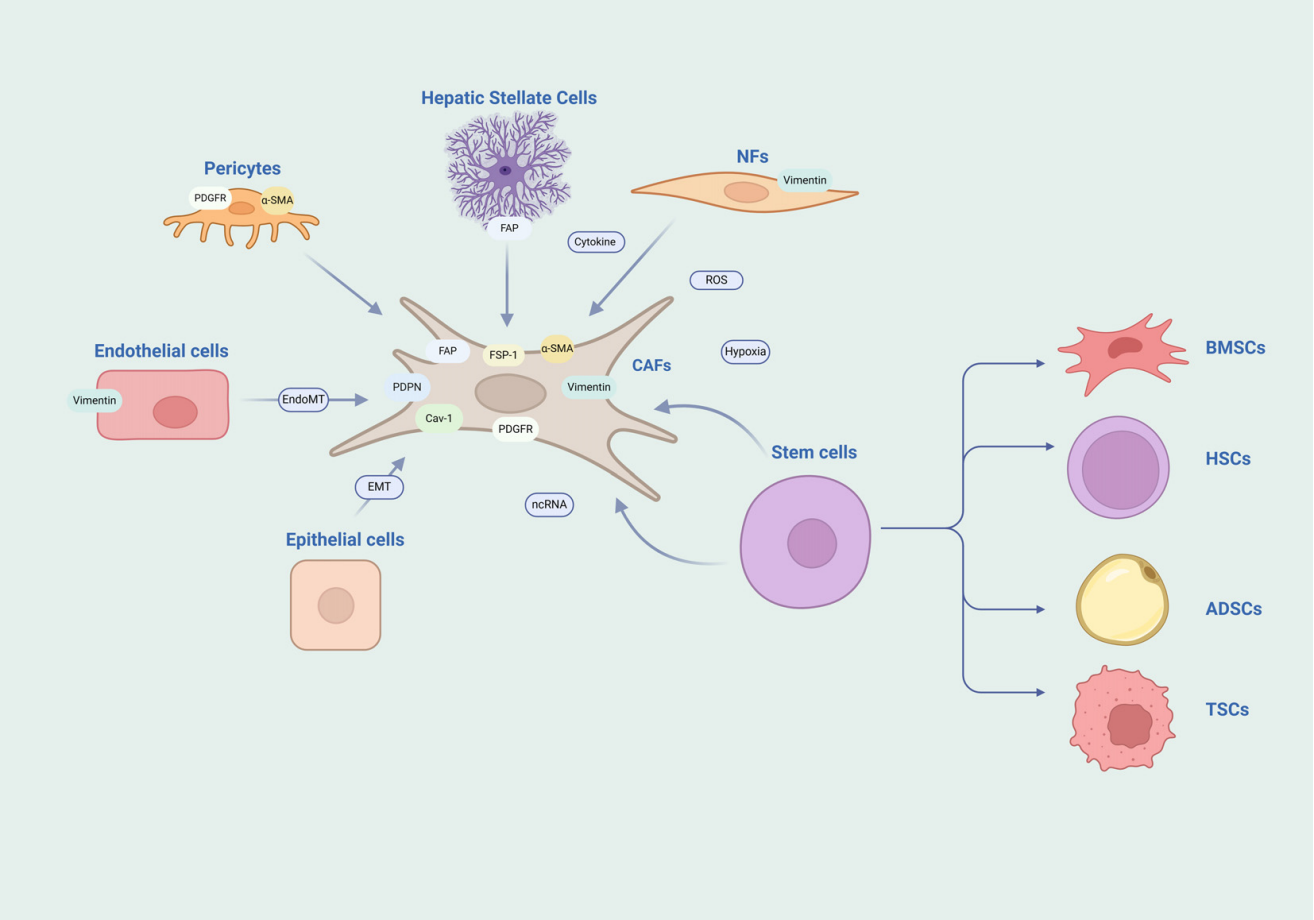
Heterogeneity of CAFs. The diversity of origins and the complexity of markers for CAFs are notable features. The heterogeneity of CAFs is highlighted by their diverse origins and the intricate array of their markers. They may arise from a spectrum of cell sources such as NFs, endothelial cells, epithelial cells, stem cells, pericytes, and stellate cells. Moreover, the markers typically associated with CAFs are not exclusive and can be found expressed in other cell types as well. NFs, normal fibroblasts; BMSCs, bone marrow mesenchymal stem cells; HSCs, hematopoietic stem cells; ADSCs, adipose derived stem cells; TSCs, tumor stem cells.

Normal fibroblasts are quiescent cells derived from mesenchymal cells that maintain tissue homeostasis and structural integrity. When fibroblasts are activated by injury or inflammatory factors, the deposition of the ECM is enhanced, and the contractile force is increased, which promotes wound healing. After the healing and remodeling are completed, fibroblasts undergo apoptosis and disappear or return to a quiescent state (10). However, if inflammation persists, such as in the case of tumors, it can lead to the emergence of a subgroup of over-activated fibroblasts. These fibroblasts, which are over-activated and maintain their functions within the tumor tissue stroma, are referred to as CAFs (11). Cytokines, reactive oxygen species (ROS), hypoxia, and ncRNAs are all key regulatory factors for fibroblast activation. A major source of CAFs is the recruitment and activation of normal fibroblasts within the local tissue (12). Stem cells retain the ability to differentiate; whether they are mesenchymal stem cells from bone marrow (13), hematopoietic stem cells (14), adipose-derived stem cells (15), or cancer stem cells (16), they can all differentiate into CAFs through signal transduction. CAFs can also originate from mature differentiated cells, such as epithelial cells that can transdifferentiate into CAFs through epithelial-mesenchymal transition (EMT), and endothelial cells that can transdifferentiate into CAFs through endothelial-mesenchymal transition (EndMT) (17, 18). Additionally, pericytes and stellate cells can also transform into CAFs (19, 20) (**Figure 1**).

### 2.2 Phenotypic heterogeneity

Pathological diagnosis remains the foundation of cancer research, and cellular origins and subtypes can be clearly identified through immunohistochemical staining and molecular analysis. However, due to the diversity of sources of CAFs, there are still no specific markers for CAFs to date. The more common markers currently used for CAFs include Fibroblast Activation Protein (FAP), Fibroblast-Specific Protein 1 (FSP1), Alpha-Smooth Muscle Actin (α-SMA), Vimentin, Platelet-Derived Growth Factor Receptor (PDGFR), Caveolin-1, and Podoplanin. However, these proteins have limitations as they are also expressed in other cells and are not specifically expressed in CAFs (21, 22). FAP is expressed in immune cells and stellate cells (23, 24); FSP1 is expressed in macrophages (25); α-SMA is expressed in pericytes and smooth muscle cells (26, 27); Vimentin is expressed in endothelial cells (28); PDGFR is expressed in pericytes (26). Therefore, the identification of CAFs is based on the strength of the expression of marker proteins, rather than the presence or absence of the protein expression, and usually a combination of multiple markers is used to determine CAFs.

### 2.3 Functional complexity

CAFs have a crucial impact on the occurrence and progression of tumors. Studies have found that most CAFs promote tumor growth, but there are also some CAFs that have anti-cancer effects. It is precisely because of the various sources and different activation mechanisms of CAFs that they exhibit a high degree of heterogeneity and have different functional effects on tumors (29). Based on biomarkers, functions, and locations, CAFs are usually divided into restraining CAFs (rCAFs), myofibroblast CAFs (myCAFs), inflammatory CAFs (iCAFs), and antigen-presenting CAFs (apCAFs) (30, 31). MyCAFs are related to the ECM and can synthesize collagen to mediate ECM remodeling; iCAFs mainly secrete various cytokines, growth factors, and chemokines to regulate tumor immune suppression; apCAFs induce naive CD4 T cells to differentiate into regulatory T cells (Tregs) in an antigen-specific manner; rCAFs are the most special and play a role in inhibiting tumor growth. MyCAFs, iCAFs, and apCAFs promote tumor cell proliferation, migration, invasion, metabolic reprogramming, therapeutic resistance, etc., through a variety of mechanisms (32, 33). Different subtypes of CAFs are not unchangeable; different subtypes of CAFs will transform into each other, indicating that multiple subtypes of CAFs will appear in the tumor tissue at the same time, and using single-cell technology, researchers have found multiple cellular phenotypic subtypes of CAFs.

## 3 Overview of long non-coding RNAs

LncRNAs lack efficient open reading frames, and only a minority can encode proteins. However, this does not imply that LncRNAs are “noise in the transcription process” (34). An increasing body of research has confirmed that LncRNAs play a crucial role in the development and progression of cancer, affecting cellular proliferation, growth, differentiation, and apoptosis, ultimately exerting either oncogenic or tumor-suppressive effects. Extensive reviews have already summarized the genomic localization, classification, functions, mechanisms of action, and tumor expression of LncRNAs (8, 35). This article will only briefly summarize their molecular functions to facilitate an understanding of how LncRNAs act as “communication mediators” between CAFs and tumor cells. LncRNAs are distributed in the nucleus, cytoplasm, and mitochondria, which endows them with the ability to interact with DNA, RNA, and proteins at multiple levels of transcriptional regulation, post-transcriptional regulation, and epigenetic regulation, exhibiting a variety of functional roles (36, 37): (1) Signal Transduction: LncRNAs are associated with specific signal transduction pathways, acting as “molecular signals” in the process of signal regulation; (2) Decoys: LncRNAs, similar to transcription factors or repressors, affect the function of regulatory factors, including enzymes, transcription factors, and miRNAs, by binding to decoy sites, thereby promoting gene activation or silencing. The competing endogenous RNA (ceRNA) network formed by LncRNA-miRNA-mRNA is a typical representative; (3) Guiding: After binding with protein complexes, LncRNAs guide them to downstream target gene promoters or genomic sites; (4) Scaffolding: LncRNAs can act as “molecular scaffolds,” linking various proteins to form complexes and directing them to specific genomic locations or target gene promoters, participating in gene activation, gene repression, and chromatin modification.

## 4 Crosstalk pathways between cancer-associated fibroblasts and tumor cells

**Figure 2:**
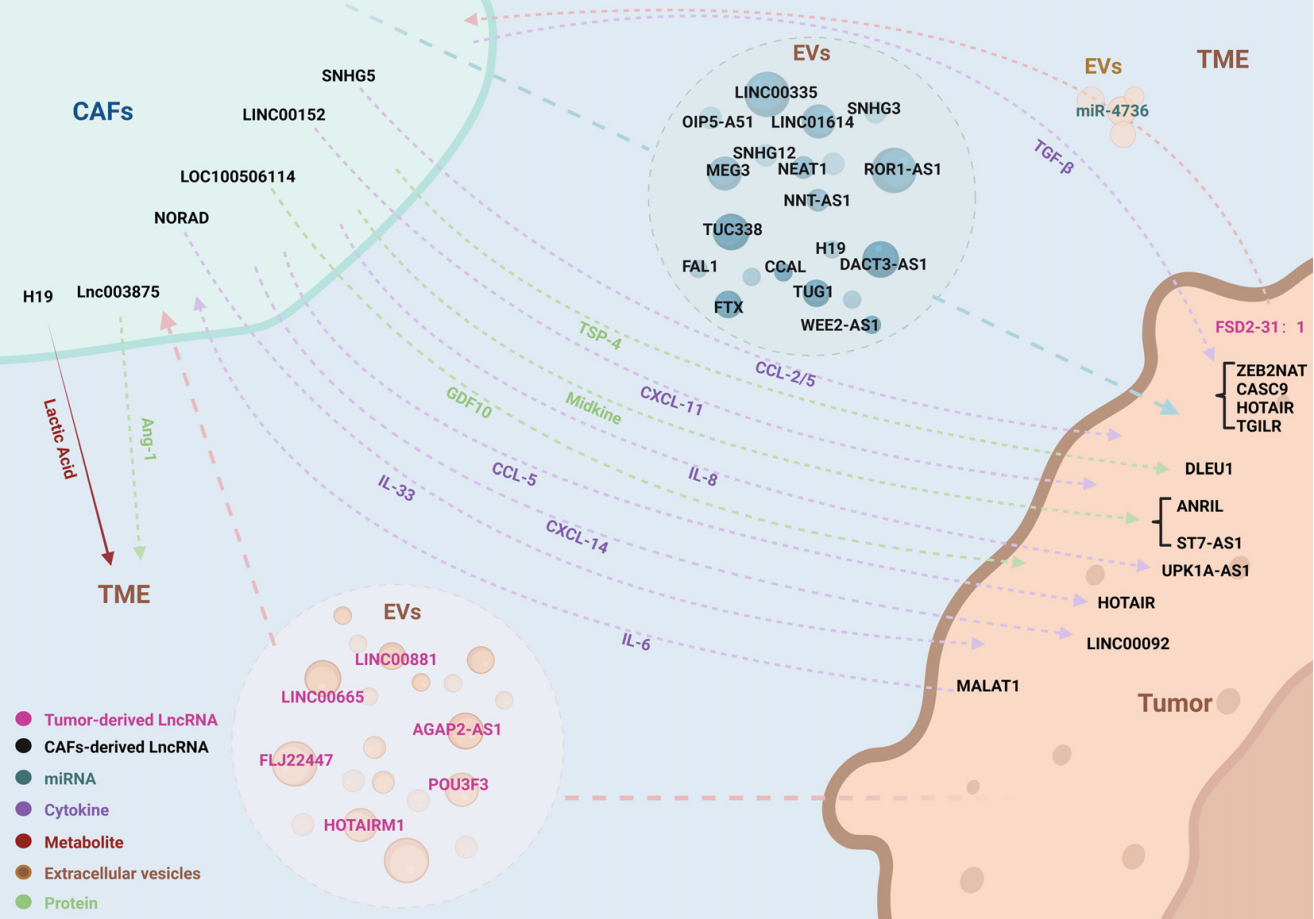
LncRNAs coordinate the complex bidirectional interactions between CAFs and tumor cells. LncRNAs orchestrate a variety of interactive pathways between CAFs and tumor cells, encompassing EVs, cytokines, secretory proteins, metabolites, and miRNAs. This communication paradigm operates in a bidirectional manner, facilitating a complex and dynamic interplay that influences tumor progression and microenvironmental modulation.

The concept of tumor cells and the TME is often metaphorically described as “seeds and soil,” highlighting the interdependence where the “seeds” cannot thrive without the nourishment and signals provided by the “soil.” This symbiotic relationship underscores the critical role of communication between the TME and tumor cells. CAFs, integral constituents of the TME, engage in diverse modes of communication with tumor cells, thereby modulating their growth. With a spotlight on LncRNAs, this review delves into the intricate pathways of interaction between CAFs and tumor cells (**Figure 2**, **Table 1**).

**Table 1:** LncRNAs mediate the communication pathways between CAFs and tumor cells.

LncRNA	Tumor	Source	Location	Medium	Function	Molecular mechanisms	Ref
LINC00355	Bladder cancer	CAFs	Tumor	Exosome	Proliferation, invasion	LINC00355/miR-15a-5p/HMGA2	(50)
ZEB2NAT	Bladder cancer	CAFs	Tumor	TGF-β	EMT	TGF-β/ZEB2NAT/ZEB2	(65)
LINC00355	Bladder cancer	CAFs	Tumor	Exosome	Drug resistance	LINC00355/miR-34b-5p/ABCB1	(74)
LINC00665	Bladder cancer	Tumor	CAFs	EVs	Lymphangiogenesis, lymphatic metastasis	LINC00665/hnRNPL/RAB27B/HGF	(95)
LINC01614	Lung cancer	CAFs	Tumor	Exosome	Energy Metabolism	LINC01614/ANXA2/P65/NF-κB/SLC38A2/SLC7A5	(82)
AGAP2-AS1	Lung cancer	Tumor	CAFs	Exosome	Tumor growth	AGAP2-AS1/EIF4A3/MyD88/NF-κB	(92)
OIP5-AS1	Lung cancer	CAFs	Tumor	Exosome	Apoptosis	OIP5-AS1/miR-142-5p/PD-L1	(84)
HOTAIR	Lung cancer	CAFs	Tumor	CCL-5	Drug resistance	CCL-5/HOTAIR/Caspase-3/BCL-2	(79)
MEG3	Lung cancer	CAFs	Tumor	Exosome	Migration, drug resistance	MEG3/miR-15a-5p/CCNE1	(62)
SNHG12	Lung cancer	CAFs	Tumor	EVs	Drug resistance	SNHG12/HuR/XIAP	(73)
HOTAIRM1	Lung cancer	Tumor	CAFs	Exosome	Migration, invasion	HOTAIRM1/miR-328-5p/SPON2	(63)
ROR1-AS1	Lung cancer	CAFs	Tumor	Exosome	Ferroptosis	ROR1-AS1/IGF2BP1/SLC7A11	(86)
TUG1	Liver cancer	CAFs	Tumor	Exosome	Migration, glycolysis	TUG1/miR-524-5p/SIX1	(61)
LINC00152	Liver cancer	CAFs	CAFs	CXCL-11	Proliferation, migration	LINC00152/miR-205-5p/CXCL-11	(58)
CASC9	Cervical cancer	CAFs	Tumor	TGF-β	Migration, invasion	CASC9/miR-215/TWIST2	(66)
LINC00881	Osteosarcoma	Tumor	CAFs	Exosome	Metastasis	LINC00881/miR-29c-3p/MMP2/NF-κB	(94)
TUC338	Laryngeal Cancer	CAFs	Tumor	EVs	Proliferation, EMT	TUC338/miR-8485/CBX2	(52)
HOTAIRM1	Glioma	Stem cell	CAFs	N/A	Cell transformation	HOTAIRM1/miR-133b-3p/TGF-β	(98)
DLEU1	Glioma	CAFs	Tumor	TSP-4	Ferroptosis	TSP-4/HSF1/DLEU1/ZFP36/ATF3	(87)
H19	Colorectal cancer	CAFs	Tumor	Exosome	Drug resistance	H19/miR-141/β-catenin	(70)
WEE2-AS1	Colorectal cancer	CAFs	Tumor	Exosome	Proliferation	WEE2-AS1/MOB1A/Hippo/YAP	(47)
FAL1	Colorectal cancer	CAFs	Tumor	Exosome	Drug resistance	FAL1/TRIM3/Beclin1	(71)
SNHG3	Colorectal cancer	CAFs	Tumor	EVs	Proliferation	SNHG3/miR-34b-5p/HuR/HOXC6	(48)
UCA1	Colorectal cancer	CAFs	Tumor	Co-culture	Proliferation, migration, EMT	UCA1/mTOR/miR-143/KRAS	(55)
CCAL	Colorectal cancer	CAFs	Tumor	Exosome	Drug resistance	CCAL/HuR/Wnt/β-catenin	(72)
FLJ22447	Oral cancer	Tumor	CAFs	Exosome	Proliferation	FLJ22447/P62/IL-33	(91)
LOC100506114	Oral cancer	CAFs	CAFs	GDF10	Proliferation, migration, EMT	LOC100506114/RUNX2/GDF10/SMAD3/ERK	(57)
H19	Oral cancer	CAFs	CAFs	Lactic acid	Tumor growth	H19/miR-675-5p/PFKFB3	(46)
ANRIL	Oral cancer	CAFs	Tumor	Midkine	Drug resistance	Midkine/ANRIL/Caspase-3/MRP1/ABCC2	(80)
FTX	Oral cancer	CAFs	Tumor	Exosome	Proliferation, migration, ferroptosis	FTX/TET2/FEN1/ACSL4	(53)
LINC00092	Ovarian cancer	CAFs	Tumor	CXCL-14	Migration, glycolysis	CXCL-14/LINC00092/PFKFB2	(67)
HOTAIR	Breast cancer	CAFs	Tumor	TGF-β	Migration, EMT	TGF-β/SMAD/HOTAIR/CDK5	(64)
SNHG5	Breast cancer	CAFs	CAFs	CCL-2/5	Angiogenesis	SNHG5/ZNF281/IGF1BP2/CCL2/CCL5/P38/MAPK	(89)
SNHG3	Breast cancer	CAFs	Tumor	Exosome	Proliferation, glycolysis	SNHG3/miR-330/PKM	(51)
H19	Breast cancer	CAFs	Tumor	EVs	Drug resistance	H19/DNMT1/miR-497	(75)
DNM3OS	Esophagus cancer	CAFs	Tumor	Co-culture	Radioresistance	PDGFβ/PDGFRβ/FOXO/DNM3OS	(77)
POU3F3	Esophagus cancer	Tumor	CAFs	Exosome	Migration, drug resistance	POU3F3/IL-6	(93)
lnc003875	Placental site trophoblastic tumor	CAFs	CAFs	Ang-1	Angiogenesis	lnc003875/miR-363/EGR1/Ang-1	(90)
DACT3-AS1	Gastric cancer	CAFs	Tumor	Exosome	Drug resistance	DACT3-AS1/miR-181a-5p/SIRT1	(76)
MALAT1	Gastric cancer	Tumor	Tumor	IL-6	Autophagy	MALAT1/ELAVL1/PTEN/AKT/mTOR/SQSTM1/NF-κB/IL-6	(97)
NORAD	Gastric cancer	CAFs	CAFs	IL-33	Proliferation, migration	NORAD/miR-496/IL-33	(56)
ST7-AS1	Gastric cancer	CAFs	Tumor	Midkine	Drug resistance, EMT	Midkine/ST7-AS1/PI3K/AKT	(68)
TGILR	Gastric cancer	CAFs	Tumor	TGF-β	Proliferation, migration	TGF-β/SMAD3/TGILR/miR-1306/miR-33a/TARBP2/TCF4	(59)
UPK1A-AS1	Pancreatic cancer	CAFs	Tumor	IL-8	Drug resistance	IL-8/NF-κB/p65/UPK1A-AS1/Ku70/Ku80	(78)
FSD2-31:1	Pancreatic cancer	Tumor	Tumor	EVs	Autophagy	FSD2-31:1/miR-4736/ATG7	(96)
NNT-AS1	Pancreatic cancer	CAFs	Tumor	Exosome	Proliferation, migration, glycolysis	NNT-AS1/miR-889-3p/HIF-1	(54)
NEAT1	Endometrial cancer	CAFs	Tumor	Exosome	Proliferation	NEAT1/miR-26a-5p/miR-26-b-5p/STAT3/YKL-40	(49)

Source, Source of LncRNAs; Location, The roles of LncRNAs are exerted at various locations; Function, The functional role of LncRNAs in tumor progression.

Extracellular vesicles (EVs) are enriched with bioactive cargo, which includes proteins, lipids, metabolites, RNA, and DNA. This cargo can be transferred to recipient cells, thereby influencing their function and facilitating intercellular communication (38). In the TME, EVs are involved in the exchange of information among various cell types, including tumor cells, epithelial cells, CAFs, endothelial cells, and immune cells, playing a crucial role in both promoting and suppressing tumor progression (39). The transfer of EVs between cancer cells and stromal cells has been identified as a mechanism for reprogramming tissues and altering environmental homeostasis. EVs derived from cancer cells, once taken up by stromal cells, can reshape the TME into a pro-tumorigenic environment; conversely, EVs derived from stromal cells can be internalized by cancer cells, further influencing tumor progression. RNA is unstable within the TME, prone to degradation by enzymes or destruction by pH changes, thus requiring the protective lipid bilayer of EVs for intercellular communication involving RNA. EVs exhibit a broad size range due to the heterogeneity in cell types and formation processes, primarily consisting of two subpopulations: exosomes, which are small membrane vesicles with a diameter of 30-150 nm, and microvesicles, which are larger membrane vesicles with a diameter of 150-1000 nm. In addition to these common subpopulations, there are also EVs with even larger diameters (40). The reciprocal exchange of EVs between CAFs and cancer cells involves the storage of LncRNA within EVs, participating in the regulation of tumorigenesis through this pathway.

Cytokines are pivotal in establishing the communication link between CAFs and tumor cells. These biological messengers carry intrinsic information, activating signaling cascades or binding to receptor proteins to engage in a variety of biological processes. The cytokine family includes growth factors, chemokines, interleukins, interferons, tumor necrosis factors, and colony-stimulating factors—glycoproteins that do not necessitate exosomal encapsulation (41). Through paracrine release, CAFs and tumor cells disseminate cytokines to transmit biological messages, modulating the expression of LncRNAs within recipient cells, thereby influencing the levels of downstream proteins and the overall trajectory of tumor progression. LncRNAs may also function upstream of cytokines, with their differential expression in CAFs and tumor cells potentially altering cytokine secretion and impacting cellular functions. Transforming growth factor β(TGF-β) is a crucial trigger for the activation and formation of CAFs and is also a participant in the malignant biological behaviors of tumor cells; this bidirectional effect is an important pathway mediated by LncRNAs for mutual communication between CAFs and tumor cells (42, 43). Furthermore, chemoattractant cytokine ligands (CCL), C-X-C motif chemokine ligands (CXCL), and interleukins (IL) have been implicated in LncRNA-mediated communication between CAFs and tumor cells via similar pathways (44). Secreted proteins, akin to cytokines, may participate in the regulatory network either upstream or downstream of LncRNAs, adding another layer of complexity to the intricate interplay between these cellular actors.

Tumors are characterized by the distinctive metabolic process of aerobic glycolysis, and CAFs play a crucial role in the intricate metabolic activities within tumors. Tumor cells induce metabolic reprogramming in CAFs, leading to an increase in glucose uptake and lactate production, which in turn supplies a carbon source for the tumor cells (45). The byproducts of energy metabolism also serve as a conduit for LncRNA-mediated communication between CAFs and tumor cells. The upregulation of LncRNAs within CAFs modifies the expression of glycolysis-related proteins downstream, enhancing lactate generation and its release into the TME, which subsequently regulates the functionality of tumor cells (46). This metabolic interplay underscores the intricate dialogue facilitated by LncRNAs, highlighting the pivotal role of CAFs in the metabolic landscape of cancer.

## 5 Long non-coding RNAs mediate the crosstalk between cancer-associated fibroblasts and tumor cells to regulate tumor growth

### 5.1 Regulate tumor proliferation

Uncontrolled proliferation of tumor cells is a defining characteristic of tumor growth. The elevated expression of LncRNAs within EVs from CAFs significantly boosts the tumor cells’ proliferative abilities upon EVs internalization. This phenomenon is exemplified in various cancers: WEE2-AS1 and SNHG3 in colorectal cancer (47, 48); NEAT1 in endometrial cancer (49); LINC00355 in bladder cancer (50); SNHG3 in breast cancer (51); TUC338 in laryngeal squamous cell carcinoma (52); FTX in oral squamous cell carcinoma (53); and NNT-AS1 in pancreatic cancer (54). The co-culture of CAFs with colorectal cells triggers an elevation in the levels of UCA1 within the tumor cells, which in turn significantly boosts their proliferative capacity (55). Within CAFs, elevated expression of NORAD augments the release of IL-33 to gastric cancer cells, thereby stimulating their proliferative activity (56). A parallel can be drawn with oral squamous cell carcinoma, where high expression levels of LOC100506114 intensify the secretion of the GDF10 protein into tumor cells (57). In liver cancer, the overexpression of LINC00152 enhances the secretion of CXCL-11 (58). Furthermore, the secretion of TGF-β by CAFs upregulates the TGILR levels within gastric cancer cells, exacerbating their proliferative capacity (59).

### 5.2 Regulate tumor metastasis

Tumor metastasis epitomizes the terminal outcome of a complex series of cellular behaviors in the invasive cascade, encompassing the spread of tumor cells to distant tissues and their adaptation to novel environments, including migration, invasion, and EMT (60). Elevated levels of LncRNAs within EVs from CAFs, once internalized by tumor cells, significantly augment their metastatic capabilities. This is exemplified in various cancers: TUG1 in liver cancer (61); MEG3 and HOTAIRM1 in lung cancer (62, 63); LINC00355 in bladder cancer (50); TUC338 in laryngeal squamous cell carcinoma (52); FTX in oral squamous cell carcinoma (53); and NNT-AS1 in pancreatic cancer (54). Co-culture with colorectal cells leads to heightened UCA1 expression, enhancing the metastatic potential (55). The secretion of TGF-β by CAFs upregulates the expression of specific LncRNAs—HOTAIR in breast cancer (64), ZEB2NAT in bladder cancer (65), TGILR in gastric cancer (59), and CASC9 in cervical cancer (66)—thus facilitating the metastatic spread of tumor cells. Furthermore, CAFs secrete a variety of cytokines and proteins that upregulate LncRNAs within tumor cells, further promoting metastasis. For instance, CXCL-14 secreted by CAFs upregulates LINC00092 in ovarian cancer (67), and Midkine upregulates ST7-AS1 in gastric cancer (68). The differential expression of LncRNAs within CAFs themselves, through the secretion of cytokines, can also foster the metastatic behavior of tumor cells. Examples include the high expression of LOC100506114 enhancing GDF10 secretion to promote oral squamous cell carcinoma metastasis (57), LINC00152 enhancing CXCL-11 secretion to promote liver cancer metastasis (58), and NORAD enhancing IL-33 secretion to promote gastric cancer metastasis (56).

### 5.3 Regulate tumor therapy resistance

Therapy resistance stands as a formidable barrier in oncology, with a range of factors—including genetic mutations, immune suppression, physical barriers, TME, and growth dynamics—propelling the development of this resistance (69). The diverse capabilities of CAFs, such as their ECM remodeling, cytokine secretion, and metabolic reprogramming, indicate an intrinsic connection to the phenomenon of therapeutic resistance. LncRNAs like H19, FAL1, and CCAL, encapsulated in CAF-derived EVs, can induce oxaliplatin resistance in colorectal cancer cells upon internalization (70–72). The internalization of CAF-origin EVs by lung cancer cells results in elevated MEG3 and SNHG12 levels, conferring cisplatin resistance (62, 73). LINC00355, transported by CAF-derived exosomes, fosters cisplatin resistance in bladder cancer cells (74), while H19 induces paclitaxel resistance in breast cancer cells through the same exosomal pathway (75). The deficiency of DACT3-AS1 in CAF-derived exosomes has been associated with oxaliplatin resistance in gastric cancer cells (76). Co-culture with CAFs leads to an increase in DNM3OS levels in esophageal squamous cell carcinoma cells, endowing them with radioresistance (77). Furthermore, cytokines secreted by CAFs, such as IL-8, which upregulates UPK1A-AS1 in pancreatic cancer, can induce oxaliplatin resistance (78). CCL-5 upregulates HOTAIR in lung cancer, causing cisplatin resistance (79). Midkine, another cytokine, upregulates ANRIL in oral squamous cell carcinoma, leading to cisplatin resistance (80), and it similarly promotes ST7-AS1 in gastric cancer, enhancing cisplatin resistance (68).

### 5.4 Regulate tumor metabolic reprogramming

Altered energy metabolism is a defining characteristic of cancer, where tumor cells undergo metabolic reprogramming to thrive and persist in inhospitable conditions, encompassing the metabolism of glucose, proteins, and lipids (81). Tumor cells exhibit a high rate of glycolysis and lactate production irrespective of oxygen availability, a metabolic pathway that is modulated by CAFs through the action of LncRNAs. For instance, TUG1 within CAF-derived EVs boosts glycolysis in liver cancer (61), while SNHG3 enhances this process in breast cancer (51), and NNT-AS1 does so in pancreatic cancer (54). The secretion of CXCL-14 by CAFs upregulates LINC00092 in ovarian cancer, thereby promoting its glycolytic activity (67). Paralleling glycolysis, the predilection for glutamine is a hallmark of tumor cells; lung cancer cells that internalize exosomes released by CAFs witness a marked elevation in LINC01614 levels, correlating with increased glutamine uptake and ATP synthesis (82). Furthermore, heightened expression of H19 in CAFs escalates lactate secretion, fostering a highly acidic tumor microenvironment that is propitious for tumorigenesis (46).

### 5.5 Regulate tumor death

Tumor cells bypass the regulatory mechanisms of the normal cell cycle, allowing for unchecked proliferation and sustaining a state of diminished apoptosis (83). CAFs secrete exosomes that introduce OIP5-AS1 into lung cancer cells, which in turn upregulates Programmed death ligand 1 (PD-L1), attenuating the incidence of apoptosis (84). Ferroptosis represents an iron-dependent form of regulated cell death, triggered by the suppression of amino acid transporters and resulting in heightened lipid peroxidation that leads to cell demise (85). Studies have revealed that CAFs modulate the susceptibility of tumor cells to ferroptosis through the action of LncRNAs. For example, FTX within exosomes derived from CAFs suppresses ferroptosis in oral squamous cell carcinoma (53), while ROR1-AS1 curbs this form of cell death in lung cancer cells (86). Additionally, the TSP-4 protein secreted by CAFs, upon internalization by glioma cells, upregulates DLEU1 expression, thereby inhibiting ferroptosis (87).

### 5.6 Regulate tumor angiogenesis

Angiogenesis is an essential condition for the formation and growth of tumors, providing vital oxygen, nutrients, and growth factors to tumor cells that fuel their swift progression. It is a well-established fact that without angiogenesis, solid tumors are unlikely to expand beyond a mere 3 millimeters (88). Within breast cancer tissues, CAFs exhibit heightened levels of SNHG5, which stimulates the secretion of CCL-2 and CCL-5 into vascular endothelial cells, thus promoting both angiogenesis and the permeability of blood vessels (89). In placental site trophoblastic tumors, CAFs demonstrate elevated expression of lnc003875, resulting in an increased release of Ang-1, a key driver of angiogenesis (90).

### 5.7 Reshape the tumor microenvironment

The interaction between tumor cells and CAFs is a two-way street. It is crucial to recognize that while CAFs can drive the biology of tumors, tumor cells can also exert influence in the opposite direction, impacting the CAF phenotype through the release of EVs, cytokines, or metabolic products. This reciprocal modulation can lead to a further reshaping of TME, ultimately fostering tumor growth. For instance, oral squamous cell carcinoma cells release exosomes rich in FLJ22447, which, upon elevating its levels in fibroblasts, activate CAFs and stimulate tumor proliferation (91). Lung cancer cells utilize exosomes to shuttle AGAP2-AS1 to fibroblasts, thereby activating CAFs and enhancing tumor growth (92). Esophageal squamous cell carcinoma cells transfer POU3F3 to fibroblasts through exosomes, activating CAFs and promoting both tumor metastasis and resistance to cisplatin (93). Osteosarcoma cells deliver LINC00881 to lung fibroblasts via exosomes, facilitating their metamorphosis into CAFs that support lung metastasis (94). Bladder cancer cells dispatch exosomes containing LINC00665 to CAFs, stimulating their activation and secretion of hepatocyte growth factor (HGF), which promotes lymphangiogenesis and lymph node metastasis (95). Pancreatic cancer cells, with diminished FSD2-31:1 expression, transport miR-4736 to fibroblasts via EVs, enhancing autophagy and curbing their activation, which in turn inhibits tumor advancement (96). An increase in MALAT1 expression in gastric cancer cells diminishes autophagy and quickens the secretion of IL-6, an activator of CAFs. Once activated, these CAFs secrete more IL-6, establishing a positive feedback loop that accelerates gastric cancer progression (97). Glioma tumor stem cells, with high expression of HOTAIRM1, upregulate TGF-β expression, driving the transformation of stem cells into CAFs and promoting tumor growth (98).

## 6 Long non-coding RNAs mediate the molecular network of crosstalk between cancer-associated fibroblasts and tumor cells

**Figure 3:**
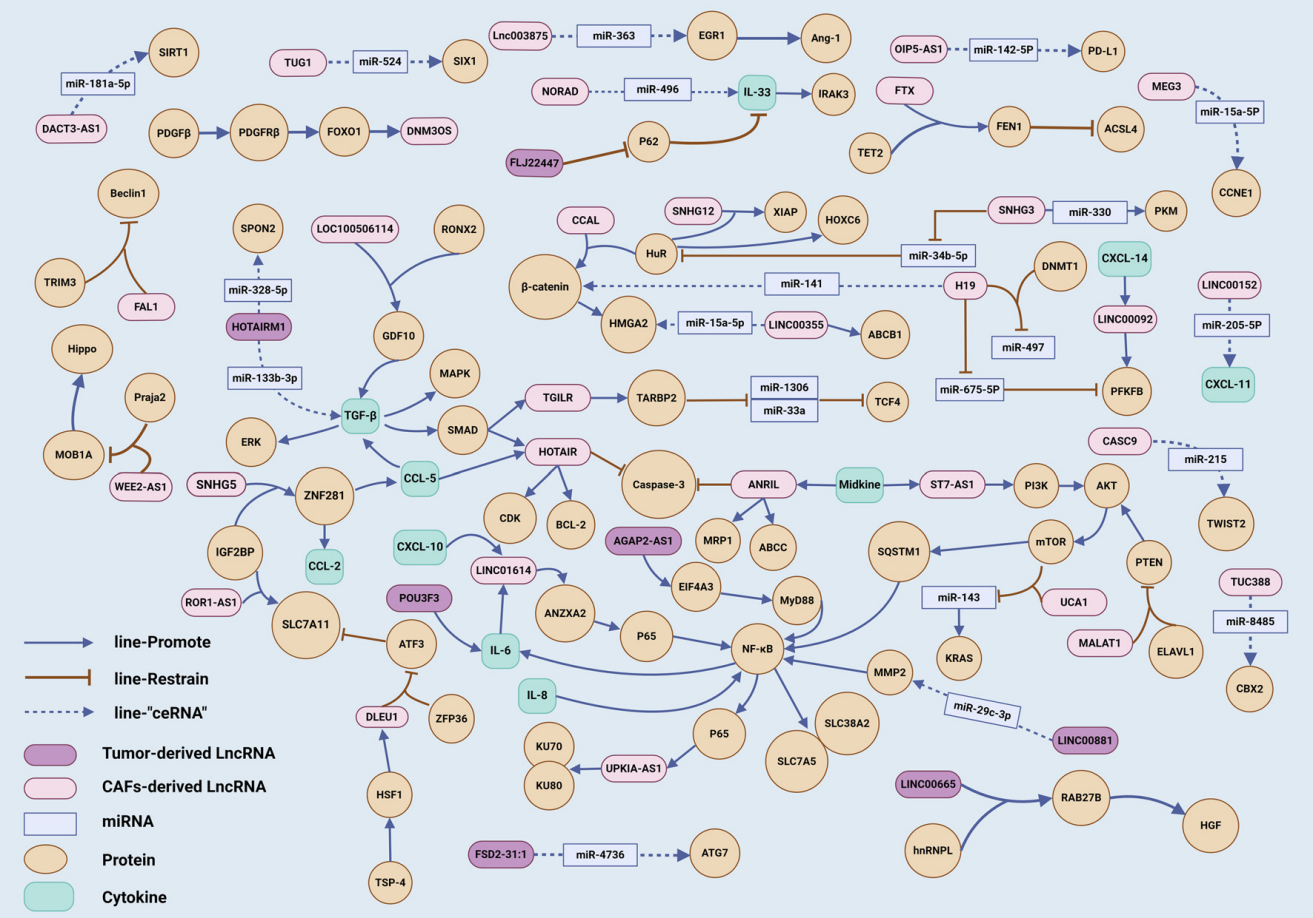
The molecular mechanism network of LncRNAs involved in the crosstalk between CAFs and tumor cells. A comprehensive network diagram was created, depicting the molecular mechanisms involving LncRNAs associated with CAFs, providing a detailed visual representation of the complex interactions and pathways in the tumor microenvironment.

Owing to the multifaceted roles of LncRNAs, they exert intricate control over downstream signaling cascades or target proteins through a spectrum of molecular mechanisms. This mediation of information exchange between CAFs and tumor cells endows LncRNAs with significant tumor regulatory capabilities. In this article, we systematically classify the associated signaling pathways and construct a molecular network map with the aspiration of pinpointing pivotal pathways. Such an approach is expected to deepen our comprehension of how LncRNAs function within the complex interplay of CAFs and tumor cells, potentially unveiling novel therapeutic targets in cancer biology (**Figure 3**).

### 6.1 TGF-β signaling pathway

The TGF-β signaling pathway exerts a multifaceted influence within TME, with its overexpression implicated in processes such as EMT, ECM deposition, the activation of CAFs, and modulation of immune responses (99). The SMAD pathway stands as a quintessential signaling cascade initiated by the intracellular kinase domain of TGF-β. Originating from CAFs, TGF-β prompts the assembly of SMAD2/3/4 onto the HOTAIR promoter, enhancing HOTAIR transcription in breast cancer cells. This triggers H3K27 trimethylation on the promoters of CDK5RAP1 and EGR-1, activating CDK5 expression and establishing the TGF-β/SMAD/HOTAIR/CDK5 axis (64). SMAD3, phosphorylated by TGF-β, translocates to the nucleus, binding to the TGILR promoter and promoting its transcription; TGILR sustains TARBP2 protein stability, hastening TARBP2-mediated degradation of miR-1306 and miR-33a, and upregulating TCF4, thereby advancing gastric cancer progression (59). LncRNAs are capable of modulating TGF-β secretion by CAFs, thereby igniting the SMAD signaling. Within CAFs, the interaction of LOC100506114 with the transcription factor RUNX2 escalates GDF10 expression. GDF10, akin to a TGF-β component, incites SMAD3 and downstream ERK protein expression, forging the LOC100506114/RUNX2/GDF10/SMAD3/ERK signaling route (57). TGF-β can also set in motion a plethora of downstream proteins via mechanisms such as phosphorylation, ubiquitination, acetylation, and protein interactions, collectively termed the non-SMAD signaling pathways (100). TGF-β from CAFs boosts ZEB2NAT transcription in bladder cancer cells, intensifying ZEB2 expression (65). In glioma stem cells, HOTAIRM1, through a ceRNA mechanism, forms the HOTAIRM1/miR-133b-3p/TGF-β regulatory loop, upregulating TGF-β to facilitate the metamorphosis of stem cells into CAFs (98). SNHG5 within CAFs catalyzes the recruitment of IGF2BP2, assembling an SNHG5-IGF2BP2-ZNF281 trimeric complex that amplifies ZNF281 and downstream CCL-2/5 expression. Subsequently, CCL-2/5 stimulates the P38/MAPK pathway associated with TGF-β in endothelial cells, thereby fostering angiogenesis (89).

### 6.2 NF-κB signaling pathway

The NF-κB signaling pathway is frequently activated in cancer and is largely attributed to the activation of inflammatory cytokines or upstream proteins within TME (101). The LINC01614, derived from CAFs, triggers P65 phosphorylation in lung cancer cells via ANXA2, thereby activating the NF-κB signaling cascade and enhancing the transcription of the glutamine transporters SLC38A2 and SLC7A5. Additionally, the secretion of IL-6 and CXCL-10 by lung cancer cells creates a positive feedback loop by further increasing LINC01614 levels in CAFs (82). IL-8 originating from CAFs initiates the NF-κB signaling pathway in pancreatic cancer cells, with P65 binding to the promoter region to boost the transcription of UPK1A-AS1, which acts as a molecular scaffold for Ku70 and Ku80, thereby augmenting the cell’s DNA repair capabilities (78). AGAP2-AS1 fosters the stabilization of its own RNA through the RNA stability protein EIF4A3 and positively modulates MyD88 and the subsequent NF-κB signaling pathway, establishing the AGAP2-AS1/EIF4A3/MyD88/NF-κB axis to stimulate CAF activation (92). LINC00881 in lung fibroblasts competitively interacts with miR-29c-3p to bind MMP2, increasing MMP2 expression and NF-κB signaling activity, thus promoting their differentiation into CAFs (94).

### 6.3 AKT signaling pathway

The AKT signaling pathway, omnipresent in cellular mechanisms, boasts an extensive array of upstream regulatory elements and a plethora of downstream signaling junctions. It plays a pivotal role in cellular processes such as survival, metabolism, and movement (102). CAFs secrete Midkine, which elevates the expression of ST7-AS1 in gastric cancer cells. ST7-AS1 facilitates the phosphorylation of PI3K and AKT, igniting the PI3K/AKT cascade and engendering drug resistance in tumor cells (68). Within the context of endometrial cancer, CAF-derived NEAT1 operates as a “ceRNA” for miR-26a/b-5p, leading to the overexpression of target genes STAT3 and YKL-40, and thus modulating tumor behavior (49). Co-culturing CAFs with colorectal cancer cells results in the joint repression of miR-143 expression by UCA1 and mTOR, causing an increase in the downstream KRAS protein levels. This interplay fosters tumor proliferation and metastatic potential (55).

### 6.4 Wnt signaling pathway

The Wnt/β-catenin signaling pathway, when dysregulated, is intimately linked to a spectrum of diseases. Activation of the Wnt pathway prompts the translocation of β-catenin to the nucleus, thereby stimulating the transcription of genes pivotal for cell proliferation, survival, and migration (103). CAFs facilitate the transfer of CCAL into colorectal cancer cells, which in turn fosters the interaction between Hu antigen R (HuR) and β-catenin, thereby igniting the Wnt/β-catenin signaling cascade (72). In the context of colorectal cancer, CAF-derived H19 also engages in a competitive binding mechanism with β-catenin to miR-141, leading to the upregulation of β-catenin and the subsequent activation of the pathway (70). Moreover, the lncRNA LINC00355, originating from CAFs, acts as a “molecular sponge,” forming the LINC00355/miR-15a-5p/HMGA2 axis and enhancing the expression of the β-catenin downstream effector HMGA2 (50).

### 6.5 Warburg signaling pathway

The Warburg effect, a quintessential characteristic of cancer, is closely linked to the activation of pathways that foster tumor metastasis and the reconfiguration of the TME (104). The augmentation of glycolysis is orchestrated through the upregulation of glycolytic enzymes and transport proteins, such as Pyruvate kinase (PK), Lactate dehydrogenase (LDH), Hexokinase (HK), Phosphofructokinase (PFK), and Glucose transporters (GLUT). SNHG3, originating from CAFs, functions as a “molecular sponge,” sequestering miR-330 and thereby enhancing the glycolytic activity in breast cancer cells by interacting with PKM (51). In a parallel mechanism, TUG1, derived from CAFs, enters liver cancer cells and engages in the TUG1/miR-524-5p/SIX1 pathway, facilitating SIX1-driven glycolysis through the “ceRNA” mechanism (61). The secretion of CXCL-14 by CAFs elevates the expression of LINC00092 in ovarian cancer cells, which in turn stabilizes PFKFB2 expression, propelling tumor glycolysis (67). The surge in glycolytic activity is predominantly governed by the modulation of Hypoxia-inducible factor 1 (HIF-1) and c-myc, with HIF-1α stimulating the expression of glycolytic enzymes like HK2 and LDHA, and transporters including MCT4 and GLUT1, thus escalating intracellular glycolysis (105). CAFs can channel NNT-AS1 into pancreatic cancer cells, establishing the NNT-AS1/miR-889-3p/HIF-1 axis that amplifies glycolytic activity and bolsters tumor progression (54). Within the TME, CAFs predominantly adopt a catabolic role, supplying lactate to tumor cells. An upregulation of H19 in CAFs diminishes the miR-675-5p-mediated degradation of the target protein PFKFB3, thereby activating PFKFB3-driven glycolysis. The heightened secretion of lactate by CAFs, in turn, accelerates the progression of oral squamous cell carcinoma (46).

### 6.6 Death receptor

The growth of a tumor signifies a perturbed equilibrium between cellular proliferation and cellular injury, yielding an array of signals within the microenvironment during cellular demise (106). For example, CAFs secrete CCL5, which elevates HOTAIR expression in lung cancer cells. HOTAIR diminishes the expression of the apoptotic initiator caspase-3 while enhancing the expression of the anti-apoptotic protein BCL-2 (79). In the context of oral squamous cell carcinoma, Midkine from CAFs boosts ANRIL expression, reduces caspase-3 levels, and stimulates the expression of MRP1 and ABCC2, thereby influencing the apoptotic landscape (80). Beyond the traditional apoptosis pathways, CAFs are instrumental in orchestrating ferroptosis, a form of regulated cell death associated with iron metabolism. CAF-derived FTX interacts with TET2, leading to the demethylation of the FEN1 promoter and an increase in FEN1 expression. FEN1, in turn, suppresses ACSL4 expression by binding to its promoter, curbing ferroptosis in tumor cells (53). ROR1-AS1, by engaging with IGF2BP1, preserves the stability of SLC7A11 mRNA, thereby inhibiting ferroptosis in lung cancer cells (86). The release of TSP-4 by CAFs instigates HSF1 activation in glioblastoma, which in turn promotes DLEU1 expression. DLEU1, through its interaction with ZFP36, facilitates the degradation of the ferroptosis transcription factor ATF3, upregulates SLC7A11 expression, and ultimately suppresses ferroptosis in tumor cells (87). DACT3-AS1, functioning as a tumor suppressor and exhibiting low expression in CAFs, fosters ferroptosis and enhances tumor cell sensitivity to oxaliplatin via the DACT3-AS1/miR-181a-5p/SIRT1 pathway (76).

### 6.7 Autophagy signaling pathway

Autophagy serves as a cellular survival mechanism, essential for the degradation of damaged cellular components and misfolded proteins, thereby nourishing cell growth. Initially, autophagy acts as a suppressor in early-stage tumorigenesis, yet it assumes a promotional role in the later stages of tumor development (107). The upregulation of the tumor-originated FLJ22447 in fibroblasts can impede the p62-driven autophagy-lysosome degradation pathway, sustaining the stability of the IL-33 protein and activating IRAK3. This sequence of events propels the metamorphosis of fibroblasts into CAFs (91). The LncRNA MALAT1, translocated from tumor cells to fibroblasts, complexes with ELAVL1, causing PTEN mRNA destabilization and the subsequent suppression of PTEN at the transcriptional tier. This cascade activates the AKT/mTOR signaling pathway, diminishes autophagic flux, and induces the overexpression of SQSTM1, which in turn activates NF-κB. These events collectively promote the activation of CAFs and the secretion of IL-6 (97). The LncRNA FAL1, emanating from CAFs, functions as a “molecular scaffold” for Beclin1 and TRIM3 within colorectal cancer cells, augmenting the ubiquitination and proteasomal degradation of Beclin1 by TRIM3 and consequently repressing autophagy (71). FSD2-31:1 exhibits tumor-suppressive effects and is underexpressed in pancreatic cancer cells. It mediates the transfer of downstream miR-4736 to fibroblasts via EVs. miR-4736 inhibits ATG7, thereby reducing autophagy levels and promoting the activation of CAFs (96).

### 6.8 HuR signaling pathway

HuR, a ubiquitous RNA-binding protein, plays a crucial role in the post-transcriptional regulation of gene expression. It is capable of responding to a multitude of stimuli, thereby facilitating the translation of specific mRNAs (108). CAFs produce SNHG3, which mitigates the degradative effect of miR-34b-5p on HuR, enhancing the stability of HOXC6 mRNA and thus fueling the proliferation of colorectal cancer cells (48). Furthermore, SNHG12 is transported to lung cancer cells from CAFs through EVs. Once bound to HuR, SNHG12 promotes the mRNA stability and expression of XIAP, contributing to the malignant progression of the disease (73).

### 6.9 Other signaling pathways

Beyond the well-documented signaling pathways like TGF-β, NF-κB, Wnt, and AKT, as previously outlined, LncRNAs also orchestrate a variety of other signaling routes between CAFs and tumor cells. In esophageal squamous cell carcinoma, for example, the PDGFβ/PDGFRβ/FOXO1/DNM3OS signaling axis (77) and the POU3F3/IL-6 pathway (93) play significant roles. The LncRNA WEE2-AS1, when transferred from CAFs to colorectal cancer cells, functions as a “molecular scaffold” for the proteins MOB1A and the E3 ubiquitin-protein ligase praja2. This interaction facilitates the ubiquitination and subsequent degradation of MOB1A, thereby dampening the Hippo signaling cascade and allowing YAP to translocate to the nucleus and initiate gene transcription (47). Furthermore, the LncRNA LINC00665, secreted by breast cancer cells, can directly interact with hnRNPL, enhancing the transcription of RAB27B. This interaction stimulates the secretion of HGF by CAFs, which in turn promotes the development of tumor lymphatic vessels (95).

In the context of lung cancer, the “ceRNA” molecular mechanism is instrumental in establishing critical pathways, including OIP5-AS1/miR-142-5p/PD-L1 (84), MEG3/miR-15a-5p/CCNE1 (62), and HOTAIRM1/miR-328-5p/SPON2 (63). Comparable “ceRNA” mechanisms have been identified in other cancers: the LINC00152/miR-205-5p/CXCL-11 pathway in liver cancer (58); the NORAD/miR-496/IL-33 pathway in gastric cancer (56); the LINC00355/miR-34b-5p/ABCB1 pathway in bladder cancer (74); the CASC9/miR-215/TWIST2 pathway in cervical cancer (66); the TUC338/miR-8485/CBX2 pathway in laryngeal squamous cell carcinoma (52); and the lnc003875/miR-363/EGR1/Ang-1 pathway in placental site trophoblastic tumor (90). Beyond their role as “ceRNA,” LncRNAs also exert epigenetic control over miRNAs. For example, H19, upon transfer from CAFs to lung cancer cells, recruits the DNMT1 enzyme, leading to increased methylation and subsequent suppression of miR-497 expression, a strategy that enhances the tumor cells’ drug resistance (75).

## 7 Long non-coding RNAs associated with cancer-associated fibroblasts serve as pivotal biomarkers for the diagnosis and prognostic assessment of tumor

Early detection of cancer is essential for precise therapeutic strategies and prognostic evaluation, but existing diagnostic approaches do not fully meet the expectations. Hence, the quest for biomarkers that exhibit both specificity and sensitivity is imperative. The dysregulation of LncRNAs is linked to the genesis, progression, metastasis, and prognostic outcomes of cancer, positioning them as promising candidates for biomarkers (109, 110). Upon reviewing the communication medium LncRNAs between CAFs and tumor cells, it has been observed that most LncRNAs are highly expressed in tumors and are associated with pathological indicators such as tumor volume, degree of differentiation, and tumor staging. Furthermore, they correlate with patient prognosis indicators, such as overall survival (OS), disease-free survival (DFS), and progression-free survival (PFS), suggesting that CAFs-associated LncRNAs may serve as potential diagnostic and prognostic biomarkers for cancer (**Table 2**). However, the current evidence is insufficient to support the use of LncRNAs as established biomarkers in clinical diagnostics. There is a need to expand sample sizes and validate these findings in large-scale, multi-population, multi-center studies. Additionally, further analysis is required to determine whether LncRNAs are independent risk factors for cancer and independent prognostic markers.

**Table 2:** The relationship between CAFs-related LncRNAs and tumor pathological indicators and prognosis.

Tumor	LncRNA	Expression level	Pathological index	Prognostic index	Ref
Lung adenocarcinoma	LINC01614	High expression	T, TNM, LNM	OS	(82)
Small cell lung cancer	MEG3	High expression	CS	N/A	(62)
Lung adenocarcinoma	HOTAIRM1	High expression	N/A	OS	(63)
Lung cancer	ROR1-AS1	High expression	N/A	OS	(86)
Colorectal cancer	H19	High expression	CS	N/A	(70)
Colorectal cancer	WEE2-AS1	High expression	Volume, TNM	OS, DFS	(47)
Colorectal cancer	FAL1	High expression	Volume, LNM, Distant metastasis	OS, DFS	(71)
Breast cancer	HOTAIR	High expression	Metastasis	OS	(64)
Breast cancer	SNHG5	High expression	TNM, LNM, Distant metastasis	OS	(89)
Gastric cancer	DACT3-AS1	Low expression	Volume, TS, LNM, Differentiation	OS	(76)
Gastric cancer	TGILR	High expression	PS, T, LNM	OS, DFS, PFS	(59)
Oral squamous cell carcinoma	FLJ22447	High expression	TNM	OS	(91)
Oral squamous cell carcinoma	H19	High expression	Meaningless	N/A	(46)
Oral squamous cell carcinoma	ANRIL	High expression	TNM, LNM	N/A	(80)
Oral squamous cell carcinoma	FTX	High expression	Differentiation, LNM, CS	OS	(53)
Esophageal squamous cell carcinoma	DNM3OS	High expression	CS	N/A	(77)
Esophageal squamous cell carcinoma	POU3F3	High expression	N/A	OS	(93)
Liver cancer	TUG1	High expression	Volume, Pulmonary metastasis, AJCC stage	OS	(61)
Liver cancer	LINC00152	High expression	N/A	OS	(58)
Ovarian cancer	LINC00092	High expression	FIGO stage, Differentiation	OS	(67)
Neuroglioma	HOTAIRM1	High expression	CS	OS	(98)
Pancreatic cancer	FSD2-31:1	Low expression	TNM, Differentiation	OS	(96)
Pancreatic cancer	UPK1A-AS1	High expression	N/A	OS	(78)
Pancreatic cancer	LINC00665	High expression	LNM	OS	(95)
Cervical cancer	CASC9	High expression	Histological type, T, LNM, FIGO stage	OS	(66)

T, T stage; TNM, TNM stage; LNM, Lymph node metastasis; OS, Overall survival; DFS, Disease-free survival; PFS, Progression free survival; CS, Clinical stage; TS, Tumor stage; PS, Pathological stage.

Liquid biopsy, as an emerging technique for tumor detection, offers several advantages, including non-invasiveness, low risk, and the ability to perform dynamic monitoring. Subsequent research is recommended to explore the potential application of CAFs-associated LncRNAs in the field of tumor liquid biopsy.

## 8 Summary and outlook

With an ever-increasing comprehension of cancer, the research spotlight has transcended the confines of the cancer cells themselves, delving into the complex functionalities of the TME, which are paramount in tumor growth, metastasis, resistance to therapy, and relapse. CAFs occupy a significant role within the TME, engaging in reciprocal communication with cancer cells to foster a nurturing environment for tumorigenesis. LncRNAs, as novel molecular couriers, have captured the interest of researchers for their multifaceted roles in the genesis and evolution of cancer.

The aberrant modulation of specific signaling cascades within tumors is a pivotal determinant of their development. CAFs or cancer cells initiate upstream signals via paracrine signaling, intercellular interactions, and exosome release, modulating the activation or suppression of particular pathways, thus influencing the malignancy of the tumor’s biological behavior. Our focus has been on the interplay between CAFs and cancer cells, encapsulating the patterns of their crosstalk and examining the signaling pathways and biological functions mediated by LncRNAs in their communication, while mapping out a molecular network based on these regulatory pathways.

Grasping the intricacies of these pathways and the modulatory influence of CAFs on tumor advancement is crucial for refining current oncological therapeutics. For example, targeting the EVs or proteins secreted by CAFs could sever the link between cancer cells and CAFs; inhibiting CAFs’ activation and reprogramming them into a dormant state of fibroblasts; or homing in on LncRNAs of CAF origin within cancer cells to impede their downstream signaling cascades. Although this review encompasses a vast array of LncRNA-mediated communication mechanisms between CAFs and cancer cells, there remains a dearth of research into the intricate regulatory networks within the TME. This gap could result in an oversight of other unknown factors or the activation of latent compensatory signals when obstructing the signaling pathways of the tumor stroma. Consequently, within the broader context of cancer treatment, it is imperative to consider the intercommunication between cancer cells and CAFs from a holistic perspective.
